# A case of Dravet syndrome with a novel SCN1A gross deletion involving the promoter region

**DOI:** 10.1038/s41439-025-00320-4

**Published:** 2025-09-03

**Authors:** Eri Nakahara Sakamoto, Shino Shimada, Tokito Yamaguchi, Tomoya Ishida, Katsumi Imai, Hidetaka Eguchi, Yasushi Okazaki, Masami Arai

**Affiliations:** 1 Department of Pediatrics and Adolescent Medicine, Juntendo University Graduate School of Medicine, Bunkyo, Japan; 2 Department of Pediatrics, NHO, National Epilepsy Center, Shizuoka Institute of Epilepsy and Neurological Disorders, Shizuoka, Japan; 3 Department of Clinical Genetics, Juntendo University Graduate School of Medicine, Bunkyo, Japan; 4 Department of Pediatrics, Sagamidai Hospital, Shizuoka, Japan; 5 Diagnostics and Therapeutics of Intractable Diseases, Intractable Disease Research Center, Juntendo University Graduate School of Medicine, Bunkyo, Japan

**Keywords:** Neurological disorders, Genetics research

## Abstract

Here we present a case of Dravet syndrome in which a novel heterozygous deletion involving the promoter region of the *SCN1A* gene was identified using next-generation sequencing and multiple ligation-dependent probe amplification. This microdeletion is believed to reduce *SCN1A* transcription, leading to haploinsufficiency. This case highlights the importance of early genetic analysis, including that of promoter regions, before the diagnostic criteria are met for the induction of specific treatments.

The *SCN1A* gene, located on 2q24.3, encodes the voltage-gated sodium channel α-subunit Nav1.1 (SCN1A) essential for generating and propagating action potential in nerve cells. This transmembrane protein forms functional channels through assembly with regulatory β1/β2 subunits, playing critical roles in channel function in inhibitory interneuron, particularly parvalbumin-positive (PV) GABAergic neuron. This cell-specific dysfunction disrupts the excitation–inhibition balance in neural circuits and induces network-level hyperexcitability. Approximately 70–80% of Dravet syndrome cases are associated with variants in the *SCN1A* gene, with more than 90% being de novo and not inherited from parents. These *SCN1A* mutations are considered loss-of-function variants associated with haploinsufficiency^1^. Loss-of-function variants in *SCN1A* cause severe epilepsy, most notably Dravet syndrome, as well as milder phenotypes such as genetic epilepsy with febrile seizures plus^2^. Recently, the gain of function of SCN1A has been linked to early infantile developmental and epileptic encephalopathy (for example, Epilepsy of infancy with migrating focal seizures (EIMFS)) with distinct features, including movement disorders. *SCN1A* variants are associated with Autism Spectrum Disorder (ASD), Sudden Unexpected Death in Epilepsy (SUDEP) and other neurological manifestations.

Dravet syndrome (OMIM: 607208) typically begins with a unilateral or generalized clonic or tonic–clonic seizure, either with or without fever, before the age of 1 year. Cognitive and behavioral impairments are often observed at the age of 2 years, and gait abnormalities such as crouch gait are usually present in the late toddler years^3^, with an overall prevalence of 6.5 per 100,000 live births^4^. Many *SCN1A* gene variants have been identified in Dravet syndrome, with over 2000 pathogenic variants of the *SCN1A* gene reported so far^5^, last evaluated on 26 May 2024^6^. The pathogenic variants primarily include single-nucleotide and multi-nucleotide variants, insertions and deletions, with a small percentage involving microdeletions. More than 50% of these mutations result in truncations, and approximately 40% are missense mutations^2,7–10^. The Human Gene Mutation Database (http://www.hgmd.cf.ac.uk/ac/index.php) includes 151 gross deletions in *SCN1A* that are relatively rare, although detailed clinical information is rarely reported. Here, we describe a novel intragenic large deletion, including the promoter region, in a patient diagnosed with Dravet syndrome at the age of 2 years and 3 months.

A 2-year-old boy was referred to the NHO Shizuoka Institute of Epilepsy and Neurological Disorders. He was the first child of a nonconsanguineous healthy Japanese parent, born at 40 weeks of gestation after a normal delivery, with a birth weight of 2740 g. The neonatal period was unremarkable. At the ages of 8 and 10 months, he experienced his first episodes of febrile focal clonic (hemiclonic) seizures, each lasting several minutes and with lateral asymmetry. First, self-limiting febrile seizures, which are common among Japanese children, were suspected, and prophylactic administration of diazepam suppositories was initiated at the age of 10 months. However, at 15 months, he experienced febrile seizures, generalized tonic–clonic seizures and prolonged seizures, which sometimes evolved into status epilepticus following fever due to infection or immunization (Fig. 1A). Mild developmental delays in language and motor skills were noted during the 18-month health checkup. He achieved independent walking ability at 18 months of age and began to speak meaningful single words at 24 months. At the age of 18 months, generalized seizures were triggered by strong light during play. After 23 months of age, he experienced recurrent afebrile focal seizures, including focal atonic and tonic seizures, with upward rolling of the eyeballs and cyanosis. Brain imaging revealed no abnormalities (Fig. 1B). An interictal electroencephalogram performed at 2 years and 2 months of age showed no abnormalities in background activity during wakefulness as well as no epileptiform abnormalities during either wakefulness or sleep (Fig. 1C). Considering that seizure triggers are common in Dravet syndrome, such as environmental heat (for example, infection, fever and immunization) and factors such as excitement and strong light, we suspected *SCN1A* haploinsufficiency, although bathing did not provoke seizures. After the initiation of valproic acid (VPA) treatment at 2 years and 3 months of age, the frequency and duration of seizures markedly reduced.

**Fig. 1:**
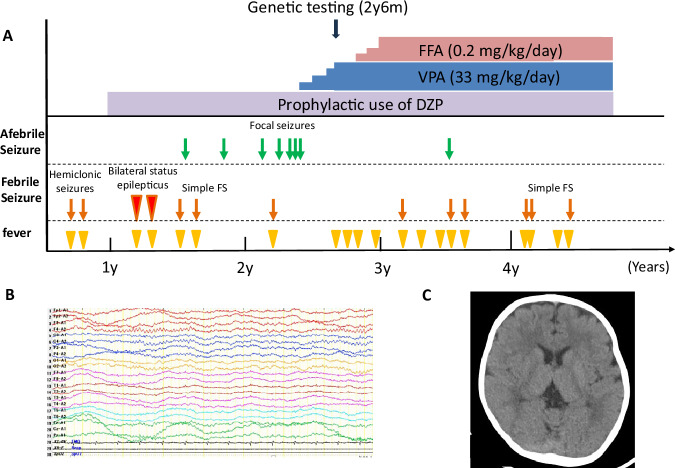
Clinical course during the treatment. **A** After the age of 2 years, the patient was started on VPA- and FFA-targeted therapy, which showed effectiveness in controlling seizures provoked by fever. **B** Plain head computed tomography at the time of admission revealed no abnormalities and calcification. **C** An interictal electroencephalogram showed no abnormalities in background activity during wakefulness as well as no epileptiform abnormalities during either wakefulness or sleep.

We provided genetic counseling to his parents and obtained written informed consent for genetic testing. Targeted panel sequencing of *SCN1A*, *SCN1B, SCN2A* and *GABRG2* genetic DNA extracted from the patient’s peripheral blood lymphocytes using the hybrid capture method was performed at the Kazusa DNA Research Institute, Chiba, Japan. No pathogenic single-nucleotide variants or small indels were detected. Quantitative analysis of the captured sequence reads suggested a possible large deletion encompassing exons 4–19 of *SCN1A* (NM001165963.4, MANE-select), although further analysis is required to confirm this finding. This study was approved by the Institutional Ethical Review Board at Juntendo University School of Medicine (E21-0153). Upon obtaining informed consent from the patient’s parent, a peripheral blood sample of the patient was provided. We then conducted multiplex ligation-dependent probe amplification (MLPA) analysis using SALSA MLPA Kit P137 Probemix (version C1) (MRC-Holland). A large deletion was identified extending from the 5′ untranslated region (2 probes, 15943-L18069 and 15942-L18068 on non-coding exon 1) to exon 19 (probe 04537L03926) in *SCN1A* (Fig. 2A, B). The *SCN1A* (NM001165963.4) consists of 28 exons, with a coding sequence initiated in exon 4. Heterozygous large deletions include the upstream enhancer/promoter regions that impact *SCN1A* protein expression and regulation, leading to reduced transcription and causing haploinsufficiency^2^. This variant in *SCN1A* was absent from controls in public databases, such as dbSNP (http://www.ncbi.nlm.nih.gov/snp/) and gnomAD (https://gnomad.broadinstitute.org/), and is not listed in ClinVar (https://www.ncbi.nlmf13.nih.gov/clinvar/) or the Human Gene Mutation Database (https://www.hgmd.cf.ac.uk/ac/index.php). Following the guidelines of the American College of Medical Genetics and Genomics^11^, the variants were classified as pathogenic (PVS1, PM2, PM4 and PP4). Parents and siblings had no history of febrile seizures and did not wish to undergo genetic testing after genetic counseling.

**Fig. 2:**
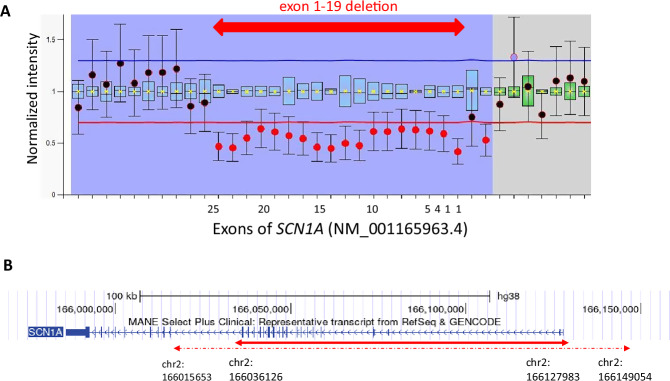
Schematic Representation of the Deleted Region in SCN1A. **A** Deletion in *SCN1A* 5′ untranslated region and exons 1–19 was detected by MLPA. **B** A schematic representation of the deleted region in *SCN1A* detected by MLPA. The maximum predicted deletion region is indicated by the dashed lines, and the minimum predicted deletion region is indicated by the solid bar. Coordinates are given according to the GRCh38 reference genome assembly.

Although the patient did not meet the clinical criteria for Dravet syndrome during infancy^12^, by the age of 2 years the typical clinical features of Dravet syndrome were evident^13–20^ (Supplementary Table 1). Based on the clinical course and genetic findings, the patient was diagnosed with Dravet syndrome because of *SCN1A* haploinsufficiency. Fenfluramine (FFA) was initiated at 2 years and 7 months of age, resulting in effective control of fever-triggered seizures. At 3 years and 2 months of age, a generalized clonic seizure lasting over 5 min occurred during a febrile episode; however, at 3 years and 4 months, no seizures were observed despite influenza infection. At 3 years and 6 months of age, the patient experienced a febrile seizure with impaired consciousness. No status epilepticus was observed in relation to febrile seizures; only brief generalized tonic–clonic seizures, consistent with typical febrile seizures, were observed around age of 4.

In general, the complete loss of expression of one *SCN1A* allele is expected to result in haploinsufficiency and may result in the most severe Dravet syndrome phenotype. However, our patient did not exhibit typical clinical features of severe Dravet syndrome in early infancy. Notably, there were no bath-induced or myoclonic seizures or seizure frequencies, with only two episodes occurring before 1 year of age. This suggests that increased expression of the remaining functional allele, potentially through mechanisms such as compensatory monoallelic expression, may have mitigated the disease severity in this case. The phenotypic heterogeneity observed in Dravet syndrome, even among patients with similar genetic backgrounds, supports variable expression and involvement of genetic modifiers.

Importantly, Dravet syndrome has specific therapeutic approaches. SCN1A-related epilepsies, particularly Dravet syndrome, have well-established therapeutic guidelines. Evidence supports the use of combination therapy with FFA, stiripentol, clobazam and VPA. Conversely, sodium channel blockers such as carbamazepine, lamotrigine and phenytoin are known to exacerbate seizure activity in these patients and should therefore be avoided. In the case of Dravet syndrome we reported, the condition was initially considered relatively mild during early childhood; however, febrile seizures recurred as the patient aged. Despite multiple adjustments in antiepileptic drug therapy, seizure control remained inadequate, highlighting the urgent need for the development of more fundamental therapeutic strategies, such as gene therapy. Early initiation of treatment, particularly aimed at preventing status epilepticus, may improve the neurological prognosis. In clinical practice, analysis methods that focus solely on exon regions may miss deletions involving promoter and intronic regions. Therefore, genetic analysis methods that include enhancer/promoter and intron regions should be generalized to clinical settings to enhance diagnostic accuracy.

## HGV Database

The relevant data from this Data Report are hosted at the Human Genome Variation Database at 10.6084/m9.figshare.hgv.3527.

## Supplementary information

SCN1A gross deletion
