# Supplementary material for: A case of Dravet syndrome with a novel SCN1A gross deletion involving the promoter region
Source: Hum Genome Var. 2025 Sep 3;12:17. doi: 10.1038/s41439-025-00320-4 (PMC12408803; doi:10.1038/s41439-025-00320-4)
Supplement: Supplementary file 1 — SCN1A gross deletion [file 41439_2025_320_MOESM1_ESM.pdf]

Supplementary Table 1 SCN1A gross deletion

Description	age of onset	syndrome	seizure type	GTCS	tonic	clonic	myoclony	atonic	absence	SE	hemiclonic	focal sz	febrile	development	Ref
296 kb incl. entire gene	under 1 year ol	Epileptic encephalopathy	Generalized tonic-clonic seizures, atypical absence seizures	+					+					mild mental developmental delay	13
entire gene	5 months	Dravet syndrome	Generalized tonic-clonic seizures, myoclonal seizures	+			+							Developmental delays and cognitive impairment from 18 months	14
36 bp, c.622_657	5 months	Dravet syndrome	Generalized tonic-clonic seizures, absence seizures, tonic seizures, myoclonic seizures, once status epilepticus	+	+		+		+	+				Developmental delays in 30 months, mild learning disorders	15
Incl. ex 2-23	ND	Dravet syndrome	12 times of epilepticus, generalized tonic-clonic seizures, focal seizures, CP, clonic seizures	+		+						+		Severe learning disabilities, developmental delay from 12 months	15
ex. 2-4,	5 years	febrile seizure	Febrile convulsions, febrile seizure+, tonic-clonic seizures	+									+	normal	16
ex. 2-4,	3 months	febrile seizure	Febrile convulsions, febrile seizure+, visual seizure									+	+	normal	16
ex. 2-4,	5 months	Dravet syndrome	Frequent febrile seizures, unilateral convulsions, absence seizures, relaxed seizures, tonic seizures, generalized tonic seizures	+	+			+	+		+		+	Autism, severe mental developmental delay	16
ex. 2-4,	4 months	febrile seizure	febrile seizure, febrile seizure +, febrile convulsions, Motion focal seizures							+		+	+	mila mental developmental delay (FSIQ=50, WISC=R)	16
ex. 2-4,	5 months	febrile seizure	febrile seizure, febrile convulsions							+			+	normal (DQ=105, GMDS-R)	16
21 bp	9 months	Dravet syndrome	Febrile seizure, hemorrhagic shock syndrome (HSES at age 4 years)										+	3 years old DQ63 (KSPD), tracheotomy and gastrostomy after HSES	17
ex. 1-20	4 months	Dravet syndrome	ND				-		-					ND	18
entire gene	4,5 months	Dravet syndrome	Febrile seizure, absence seizure, focal seizure, myoclonus				+		+			+	+	ND	19
ex. 2-6	9 months	Dravet syndrome	Febrile seizure, febrile convulsions, tonic-clonic seizures, myoclonus, CP	+			+			+		+	+	severe mental developmental delay	19
ex. 3	6 months	Dravet syndrome	Febrile seizure, tonic-clonic seizures, myoclonus, CP	+			+					+	+	Moderate mental developmental delay	19
ex. 2-16, c.365-?_3396+?del	8 months	Dravet syndrome	Focal seizures, convulsantis, febrile seizure									+	+	severe mental developmental delay	20
Total deletion of 73831bp containing putative promoter regions upstream of SCN1A 5` non-coding exons	6 months	Dravet syndrome	Tonic seizures, myoclonus, absence seizures, focal seizures, convulsantis, febrile seizures	+			+		+	+		+	+	Moderate mental developmental delay	20
